# Reply to: “Malignant transformation of hepatocellular adenoma”

**DOI:** 10.1016/j.jhepr.2022.100429

**Published:** 2022-01-08

**Authors:** Sophie Chopinet, Aurélie Beaufrère, Olivier Soubrane, François Cauchy, Valérie Paradis

**Affiliations:** 1 Department of HPB and Liver transplantation, Assistance Publique-Hôpitaux de Paris and Université de Paris, Hôpital Beaujon, Clichy, France; 2 Department of pathology, Assistance Publique-Hôpitaux de Paris and Université de Paris, Hôpital Beaujon, Clichy, France

To the Editor:

We are honored to receive the pertinent and relevant comments of Céline Julien *et al.*,^1^ and we enjoyed reading about the Bordeaux group’s experience with malignant transformation of hepatocellular adenoma (HCA). We are pleased to address the issues they raised regarding our study, which concerns a matched case-control study comparing the long-term outcome after liver resection for HCA with malignant transformation (MT-HCA) to hepatocellular carcinoma (HCC) developed on normal liver-parenchyma.^2^

First, we agree that the long study period between 2001-2019 led to biases especially regarding progress made on MRI, HCA classification and molecular analysis. Imaging modalities and perioperative management dramatically changed, which may have led to substantial biases in the imaging interpretation, with a preoperative MRI missing in 7 cases (18%). While significant progress has been made over the last two decades, particularly in the diagnosis of HCA subtypes on MRI, only two subtypes are well-recognized, HNF1α-mutated HCAs (H-HCA) and inflammatory HCA (I-HCA), and no specific imaging pattern has been identified for the diagnosis of β-catenin subtype (β-HCA) and MT-HCA.^3^ Since the original description of the HCA classification,^4^ progress has been made and new subtypes have been described. Thus, for this reason, shHCA, a rare subtype (<5%) recently described in 2017^5^ was not classified in our study. In our study, 8 MT-HCA from 40 cases did not have extensive molecular analysis because liver resections were performed in the early 2000s. In these cases, the subtype classification was based on morphological and immunohistochemical features, which have been nicely described by Bioulac-Sage *et al.*^6^

Second, small foci of malignant transformation HCA (SF-HCA) were defined by the presence of foci of malignancy <1 cm within an HCA and malignant HCA (M-HCA) by the presence of macroscopic malignant HCC nodules larger than 1 cm. The diagnosis of malignant transformation of HCA was achieved on surgical specimens by pathological analysis, and all cases included in this study displayed clear foci of malignant transformation based on cytological and architectural features, including reticulin staining in all cases and immunophenotypical markers (such as Glypican-3) when necessary. Borderline HCA without overt foci of malignant transformation were excluded from the study mainly because of the lack of consensual definition in the literature, and variability in interpretation between pathologists. We acknowledge that the distinction of borderline HCA from degenerated HCA or from a well-differentiated HCC may in some cases be difficult to do.^7^ Molecular analysis of *TERT* promoter mutations, as a molecular marker of malignancy, may be helpful in these cases. For instance, *TERT* promoter mutations have been reported in only 17% of borderline lesions compared with 60% of HCC.^8^^,^^9^

Third, Céline Julien *et al.*, noted that I-HCA was the main subtype in our study, indeed, I-HCA was the main subtype (47% of cases) followed by β-HCA (30%) and β-IHCA in 10%. It is well-admitted that risk of malignant transformation is associated with large tumor size (>50 mm), male sex, and presence of βex3 mutations. The risk of malignant transformation into HCC may reach 40% in β-HCA, an incidence higher than for other molecular HCA subtypes.^5^ Thus, our high proportion of β-HCA or β-IHCA (40%) is in accordance with the literature. I-HCA represents the major subtype in surgical series,^10^ more often resected than H-HCA due to the risk of malignant transformation and the potential association with β-catenin mutations. Moreover, I-HCA resected in our study were large tumors, with a mean size of 9.2 cm (5.5-16), all of which were larger than 5 cm.

As mentioned by Céline Julien *et al.*, HCCs can frequently overexpress inflammatory proteins and a low level of LFABP, which can be misleading. In addition to suggestive morphological features, I-HCA were confirmed by immunohistochemical stainings with serum amyloid A (considered positive when at least 10% of tumoral hepatocytes displayed cytoplasmic staining) and C-reactive protein (considered positive in case of diffuse and intense pattern). To avoid confusion with well-differentiated HCC, all slides of the tumor were carefully reviewed by a pathologist experienced in liver pathology.

In our study, 10 patients experienced recurrence, among them, 4 patients had satellite nodules and 2 had microvascular invasion, but none had poorly differentiated HCC.

Finally, we totally agree with Céline Julien *et al.* that long-term outcomes are relevant to improve management strategies of malignant transformation risk in HCA, and that specific adjusted risk factors of malignant transformation for each patient with HCA should be evaluated and should help to determine the most appropriate management.

## Financial support

The authors received no financial support to produce this manuscript.

## Authors’ contributions

SC, AB, VP wrote the manuscript. FC, OS reviewed and corrected the data and manuscript.

## Conflict of interest

The authors declare no conflicts of interest that pertain to this work.

Please refer to the accompanying ICMJE disclosure forms for further details.
